# Prevalence and determinants of the dangerous selfie among medical and nursing students: a cross-sectional study from eastern India

**DOI:** 10.1186/s12889-020-08785-4

**Published:** 2020-05-06

**Authors:** Priyamadhaba Behera, Arvind Kumar Singh, Vikas Bhatia, P. S. Preeti, Rishav Kumar, Satyajeet Das, Rupesh Tholia, Ritajyoti Ghosh, Sandeep Kumar, K. S. Safiya, Rojismita Purohit, Raman Bansal

**Affiliations:** Department of Community Medicine and Family Medicine, AIIMS, Bhubaneswar, 751019 India

**Keywords:** Selfie, Dangerous selfie, Selfitis behaviour scale, Medical and nursing students, India

## Abstract

**Background:**

Globally, there has been an exponential rise in smartphone use and selfie taking among youth. To make selfies exciting, dangerous selfies are often taken that may lead to catastrophic consequences, including death. This study aims to estimate the prevalence of dangerous selfies and to determine the factors associated with dangerous selfies among medical and nursing students in India.

**Methods:**

The study was conducted at the All India Institute of Medical Sciences (AIIMS), Bhubaneswar, India, in April–August 2018. The inclusion criteria were students enrolled in the Bachelor of Medicine and Bachelor of Surgery (MBBS) and nursing courses of AIIMS, Bhubaneswar. Students who did not use smartphones were excluded from the study. The interview schedule and Selfitis Behaviour Scale (SBS) were used to collect information on sociodemographic variables, smartphone use and variables related to selfies and dangerous selfies. Forward stepwise logistic regression was undertaken with the probability of entry and removal as 0.05 and 0.10, respectively.

**Results:**

Of 633 eligible participants, 595 were included in the study. The mean (SD) age of the participants was 21.2 (1.6) years. More than half (56.8%) of the participants were female, 384 (64.5%) were medical students and 211 (35.5%) were nursing students. Nearly two-thirds of the participants (70.6%) preferred to take selfie. One hundred thirty three (40.3%) of the participants posted selfies on social media daily. The prevalence of dangerous selfies was 8.74% (95% CI: 6.73–11.28). Eight injury episodes while taking selfies were reported by seven (1.2%) participants. Being male (AOR 4.96, 95% CI 2.53–9.74), posting selfies on social media daily (AOR 3.33, 95% CI 1.71–6.47) and an SBS score > 75 (AOR 4.97, 95% CI 1.43–17.28) were independent predictors of dangerous selfies.

**Conclusion:**

Nearly one in ten medical and nursing students reported having taken a dangerous selfie, and one in one hundred reported having been injured while attempting to take a selfie. Being male, posting selfies on social media daily and an SBS score > 75 were independent predictors of dangerous selfies. Further research is required to identify the community burden of dangerous selfies and to develop strategies to prevent selfie-related fatalities among youths.

## Background

The present age can undoubtedly be branded as the era of advancements in mobile technologies. There are approximately 2.5 billion smartphone users worldwide and 650 million users in India [1]. In the last three decades, advancements in mobile technology have led to new features, such as the selfietool, online gaming, gambling, and shopping [2]. The selfietool, in particular, has become a trend and a medium of self-representation, especially among young people.

The Oxford English Dictionary defines a “selfie” as a photograph that one has taken of oneself, generally with a smartphone or a web camera, that is often uploaded to a social media platform [3]. The word selfie has become so common that the Oxford English Dictionary declared it “the Word of the Year” in 2013 [4]. According to a study by Lee and Sung (2016), smartphone users take approximately 93 million selfies each day [5].

According to the “Mobile Technology and Home Broadband 2019” survey performed by the Pew Research Center, 96% of those aged between 18 and 29 years in the U.S. owned a smartphone [6]; another study estimated that 98% of the participants (aged 18 to 24) took selfies, and 69% tended to share selfies 3 to 20 times daily [7]. A similar study conducted among students in the city of Mumbai revealed that 42.6% regularly took selfies, while 18.1% of girls and 15.2% of boys took an average of more than four selfies per day [8]. A survey by Era Dutta et al. reported that the prevalence of “addiction to selfie taking” was 13% among adolescents in Mumbai [8]. A cross-sectional study conducted among medical and nursing students in Bangalore, India, estimated that the majority of students took selfies, and nearly one-quarter of the students had a condition termed selfitis (the obsessive taking of selfies) [9]. Certain studies have revealed that the habit of taking selfies can be linked to grandiosity, narcissism and dysmorphic disorder [10]. Currently, to add excitement to their selfies and to portray themselves as what young people deem “cool,” people are increasingly taking selfies in situations that can be potentially dangerous to gain attention on social media [11, 12]. These situations may lead to fatal consequences. There are various apps and blogs that challenge people to take selfies in difficult situations, for example, underwater selfie challenges. People often risk their lives to respond to these challenges, which can be potentially harmful [13, 14]. Such risks can be observed by looking at images posted even in the face of natural disasters, when people should be protecting themselves and helping others. According to a BBC report published on 4 October 2018, 259 people died while taking selfies in 2018 alone [15]. The deaths of Gavin Zimmerman (New South Wales, Australia) [16] and Tomer Frankfurter (Yosemite National Park) [16] brought this issue, which had previously been unheard-of, into the limelight. Selfie deaths are often underreported or not reported as the official cause of death. The highest number of selfie injuries and deaths has been reported in India, accounting for approximately 50% of the total selfie deaths reported worldwide, followed by Russia, the United States, and Pakistan [17]. The ratio of casualties to incidents in India is double that in other countries [17] Three out of four selfie deaths occur in the youngest age group, less than 25 years [18]. There has been an exponential rise in selfie taking among youths as well as among medical and nursing students in India [9, 19]. However, the burden of dangerous selfies and the associated risk factors remains unexplored in the literature. This study aims to estimate the prevalence of dangerous selfies and to determine the factors associated with dangerous selfies among medical and nursing students in India.

## Methods

### Participants

We conducted a cross-sectional study among medical and nursing students of the All India Institute of Medical Sciences (AIIMS) (hereafter referred to as ‘the institute’) in Bhubaneswar, Odisha, India, in April–August 2018. AIIMS Bhubaneswar was chosen for the study setting because, as a central institute, it has student representatives from all over India, and almost all the students have access to a smartphone, thus ensuring the feasibility of the study. The inclusion criteria were students enrolled in the Bachelor of Medicine and Bachelor of Surgery (MBBS) and nursing courses at the institute. Students who did not use smartphones were excluded from the study. There were no available prevalence studies regarding dangerous selfie behaviour among young adults. We used the prevalence of addiction to selfies as a proxy for the act of taking a dangerous selfie and estimated the sample size accordingly. Taking the prevalence as 13% [8], alpha = 5%, beta = 20%, the suitable sample size was calculated to be 643.

### Measures

In our study, a selfie is defined as self-portrait photography of oneself (or oneself with other people) taken with a camera or a camera phone held at arm’s length or pointed at a mirror and usually shared through social media. Social media is defined as websites and applications, such as Facebook, Twitter, WhatsApp, and Instagram,that enable users to create and share content or to participate in social networking. A dangerous selfie is defined as a selfie taken in a situation that is potentially dangerous to oneself and others in the vicinity. These situations include posing amid heavy traffic while driving, while diving, with a vicious animal (wildlife selfie), at high-altitude edges (such as mountaintops and cliffs) or on megastructures, during natural disasters, and in front of burning buildings. A structured interview schedule was developed based on a review of the literature.

The interview questions were pilot-tested among 20 students and then finalized. The interviews were used to collect information on sociodemographic variables, smartphone use and variables related to selfies and dangerous selfies. Participants were considered to take selfies daily and post selfies on social media daily if they had done so for the previous15 days or more. The dangerous selfies was considered when the participants had ever taken one. The Selfitis Behaviour Scale (SBS) is a validated tool for the assessment of selfie-related behaviour. The scale was developed by Balakrishnan & Griffiths [20]. It has six components: environmental enhancement, social competition, attention-seeking, mood modification, self-confidence, and social conformity. It consists of 20 items. The domains of environmental enhancement and social competition each contain four items, and the domains of attention-seeking, mood modification, self-confidence, and subjective conformity each contain three items. For example, “Taking selfies gives me a good feeling to better enjoy my environment” is related to assessing environmental enhancement. Similarly, “Taking different selfie poses helps increase my social status” is related to social competition. The response to each item was rated on a 5-point Likert scale (1 – strongly disagree; 2 – disagree; 3 – neither agree nor disagree; 4 – agree; 5 – strongly agree). The total scores ranged from 20 to 100. The behaviour was categorized as normal, borderline, acute and chronic selfitis with scores ranging from 20 to 40, 40 to 60, 60 to 80, and 80 to 100, respectively [9].

### Procedures

Ethical approval was obtained from the Institutional Ethics Committee, All India Institute of Medical Sciences, Bhubaneswar (Ref Number: T/IM-NF/CM&FM/17/47). Permission was received from the dean of the institute, and all medical (400) and nursing students (235) were contacted. The purpose of the study was explained to the students through participant information sheets. Written informed consent was obtained from participants above 18 years of age. Written informed assent was obtained who were between 17 and 18 years of age. Written informed consent was also obtained from the dean of the institute. The Institutional Ethics Board approved this consent procedure. Then, the structured interview schedule and SBS were privately administered to the medical and nursing students in the group. Two additional visits were made to the student hostels to contact participants who were absent during the first visit. The participants who were absent even after the three visits (made 1–2 weeks apart) were categorized as non-respondents and excluded from the study. The participants who had positive scores for dangerous selfies were referred to the psychiatric clinic.

### Data analysis

The data were entered into Excel. The analysis was performed in SPSS 16.0. The results were reported as proportions with a 95% confidence interval (95% CI) for the reported dangerous selfies. The mean (SD) was reported for continuous variables. Univariate and multivariate analyses were performed to examine the association of variables with dangerous selfies. The strength of association was measured as an odds ratio. A value of *p* < 0.05 was considered significant. Logistic regression was performed for the variables age, gender, occupation, taking selfies on a daily basis, posting selfies on social media daily and SBS score. Unadjusted odds ratios and *p*-values were reported in univariate analyses. Multivariate analysis was performed for independent predictors of dangerous selfies. The variables for which *p* < 0.25 in the univariate analysis were included in the multiple logistic regression. Forward stepwise logistic regression was undertaken with the probability of entry and removal as 0.05 and 0.10, respectively [21].

## Results

There were a total of 635 participants. Eighteen participants did not give consent, and 20 participants could not be contacted because of their absence despite three visits (one private visit in the group and two additional visits to the hostels) 2–4 weeks apart. Of 597 participants who were interviewed, two were excluded because they did not use smartphones. Finally, of 633 eligible participants, 595 students participated in the study, and the response rate was 94%. Of the 595 participants, 384 (64.5%) were medical students, and 211 (35.5%) were nursing students (Table 1). The mean (SD) age of the participants was 21.2 (1.6) years. The majority of the participants (91.4%) were between 18 and 22 years old, and more than half (56.8%) were females. Nearly two-thirds of the participants (70.6%) took selfies. Among them, 78.6% took selfies daily, 15.5% at least once a week and 5.9% only on special events/occasions.

**Table 1:** Socio-demographic characteristics of the sample (*n* = 595)

**Characteristics**		**Frequency**	**%**
Age (in completed years)	17–19	199	33.4
	20–22	345	58.0
	≥23	51	8.6
Gender	Female	338	56.8
	Male	257	43.2
Course of study	Medical students	384	64.5
	Nursing students	211	35.5

The average number of selfies taken per day by a participant was 3.6. The average number of selfies posted by a participant per day was 1.35. The majority (90.9%) of those who took selfies daily took 1 to 4 selfies a day, while 1.2% took more than eight selfies a day. Of the 330 participants who took selfies daily, 133 (40.3%) uploaded selfies on social media daily. One hundred twenty-seven participants (38.4%) posted selfies on social media one to three times a day, while six participants (1.8%) posted selfies on social media more than three times a day (Table 2). Approximately 8.74% (95% CI: 6.73–11.28) of the participants admitted taking dangerous selfies. Eight injury incidents while taking selfies were reported by 7 (1.2%) participants (one participant had been injured twice).

**Table 2:** Distribution of participants by taking Selfies

**Characteristics of participants**		**Frequency**	**%**
Do you take selfies? (*n* = 595)	Yes	420	70.6
	No	175	29.4
Do you take selfie daily or weekly or on special event/occasion only? (*n* = 420)	Daily	330	78.6
	Weekly	65	15.5
	On special event/occasion only	25	5.9
Number of selfies taken per day (*n* = 330)	1 to 4	300	90.9
	5 to 8	26	7.9
	> 8	4	1.2
Number of selfies posting on social media daily (*n* = 330)	None	197	59.7
	At least One time to three times	127	38.5
	More than three times	6	1.8

The mean (SD) SBS score was 47.1 (14.9) (Supplementary Table 1). Based on the SBS score, nearly half of the participants (52.6%) were classified as borderline; however, 8 (1.3%) participants had chronic selfitis, and 109 (18.3%) participants had acute selfitis (Table 3). The highest quartile (SBS score > 75) was associated with dangerous selfies in the univariate analysis (Supplementary Table 2).

**Table 3:** Distribution for participants by Selfitis Behaviour Scale (SBS) score

**Classification of Selfitis by SBS score**	**Male n (%)**	**Female n (%)**	**Total n (%)**
Normal (20–39)	90 (35.0)	75 (22.2)	165 (27.7)
Borderline (40–59)	129 (50.2)	184 (54.4)	313 (52.6)
Acute Selfitis (60–79)	33 (12.8)	76 (22.5)	109 (18.3)
Chronic selfitis (80–100)	5 (2.0)	3 (0.9)	8 (1.3)
	**257 (100)**	**338 (100)**	**595 (100)**

Dangerous selfies were more prevalent in the 17–22 age group (9.0%) than in the ≥23 age group (5.9%), among males (14.4%) than among females (4.4%), among medical students (11.2%) than among nursing students (4.3%), among participants who took selfies on a daily basis (11.2%) than among those who did not take selfies on a daily basis (5.7%), among participants posting selfies on social media daily (15.0%) than among those not posting selfies on social media daily (6.9%) and among participants with an SBS score > 75 (35.7%) than among those with an SBS score ≤ 75 (8.1%) (Table 4). Logistic regression analysis showed that being male (AOR 4.96, 95% CI 2.53–9.74), posting selfies on social media daily (AOR 3.33, 95% CI 1.71–6.47), and an SBS score > 75 (AOR 4.97, 95% CI 1.43–17.28) were independent predictors of dangerous selfies (Table 4). Occupation (OR 2.83, 95% CI 1.35–5.93) and taking selfies on a daily basis (OR 2.11, 95% CI 1.13–3.93) were associated factors in the univariate analysis; however, these factors were not significant after adjusting for other factors in the multivariate forward stepwise logistic regression (Table 4).

**Table 4:** Associated risk factors for “Dangerous Selfie”

**Variables**	**Total number of participants (*n* = 595)**	**Total number of participants “took selfie in a situation which could be potentially dangerous”*n*** **= 52 (%)**	**Unadjusted odd ratio (95% CI)**	***P*-Value**	**Adjusted odd ratio (95% CI)**	***P*-value**
**Age (in years)**						
17–19	199	18 (9.0)	1.59 (0.45–5.63)	0.464		
20–22	345	31 (9.0)	1.58 (0.47–5.37)	0.471		
≥ 23	51	3 (5.9)	Reference			
**Gender**						
Female	338	15 (4.4)	Reference		Reference	
Male	257	37 (14.4)	3.62 (1.94–6.76)	< 0.001	**4.96 (2.53–9.74)**	**< 0.001**
**Occupation**						
Nursing students	211	9 (4.3)	Reference			
Medical students	384	43 (11.2)	2.83 (1.35–5.93)	0.006		
**Taking selfies on a daily basis**						
No	265	15 (5.7)	Reference			
Yes	330	37 (11.2)	2.11 (1.13–3.93)	0.019		
**Posting selfies on social media daily**						
No	462	32 (6.9)	Reference		Reference	
Yes	133	20 (15.0)	2.38 (1.31–4.32)	0.004	**3.33 (1.71–6.47)**	**< 0.001**
**Selfitis Behaviour Scale (SBS) score**						
SBS score (0–75)	581	47 (8.1)	Reference		Reference	
SBS Score (76–100)	14	5 (35.7)	**6.31 (2.03–19.60)**	**0.001**	**4.97 (1.43–17.28)**	**0.012**

## Discussion

The first known selfie was taken in 1839 [22]. However, in recent years, taking personal or group selfies has become very popular among adolescents and young people [23]. Medical and nursing students are no exception. With the increase in the need to make selfies exciting and to gain attention on social media, dangerous selfies are becoming increasingly common [18]. The mass media have reported many incidents of injuries and even fatalities while taking selfies, but the scientific literature has not sufficiently explored the topic. In our study, we found that nearly one in ten participants had taken dangerous selfies, and one in one hundred participants had injured themselves while taking selfies. Temporary distraction while taking a selfie reduces a person’s situational awareness, which increases risky behaviour, as the person loses the sense of danger in such situations [11, 24]. This may explain selfie-related injuries and fatalities. Therefore, preventing dangerous selfies remains vital to avoid selfie-related injuries and fatalities.

Selfitis (the obsessive taking of selfies) as a disorder has recently attracted the attention of researchers. Nearly one-fifth of the participants in our study had selfitis. Previous studies have shown that selfitis is linked to poor work/academic performance, peer pressure, unwanted stress, unhealthy family relations, conflicts, and other problems. It may lead to complications such as low back pain, cervical spondylitis, awkward posture, frozen shoulder, and tennis elbow [25].

In our study, being male was a risk factor for dangerous selfies. A study in Poland by Sorokowski P et al. reported that narcissism was associated with men posting selfies online more than women [26]. Previous studies indicate that self-presentation, the demand for admiration, and leadership associated with narcissism among men are reasons for posting different types of selfies, including dangerous selfies [5, 27]. A comprehensive study of worldwide selfie-related accidental mortality revealed that 82% of the victims were male [18]. Similarly, a study in India by Bansal A et al. reported that 72.5% of selfie deaths occurred among males [17], which also supports our study finding that being male is a risk factor for dangerous selfies. Impulsive action is typically greater in males than females [28]. This may be another potential reason why male sex was a risk factor for dangerous selfies.

Posting selfies on social media daily was another risk factor for dangerous selfies. Although taking selfies daily was not a risk factor for dangerous selfies, those who posted selfies daily had taken dangerous selfies. The possible link is that the appreciation associated with ‘likes’, ‘followers’ and positive comments on social media is rewarding for people with higher levels of loneliness, isolation, and insecurity [29]. Attention-seeking, communication, archiving and entertainment are the motivations for posting selfies on social media [30]. Our findings that taking too many selfies and posting them online are causes of selfie-related injuries and fatal events were consistent with the literature [18].

Based on the categories in our study, a high SBS score (highest quartile, > 75) was an independent predictor of dangerous selfies. The SBS has six components: environmental enhancement, social competition, attention-seeking, mood modification, self-confidence, and social conformity. No previous research has considered the association of the SBS score and dangerous selfies. Thus, our study suggests that the SBS may serve as a screening tool to identify the probability of dangerous selfies; however, more research is required to further delineate the role of the SBS. Selfies in themselves are not harmful, but the human behaviour that accompanies selfies can be dangerous. When taking a selfie, a person needs to ensure that he or she is in a safe place and that his or her life is not in danger. Concerns regarding selfie injuries and fatalities have already led to various actions. The government of Russia has released a full-scale marketing campaign with icons referring to dangerous selfies [31], and selfies have not been allowed in the Hong Kong Marathon since 2014 [32]. Taking dangerous selfies and posting dangerous selfies on social media should be restricted. Lack of situational awareness and over-enthusiastic behaviour plays a vital role in selfie-related injuries. Hence, the implementation of “no selfie zones” in high-risk areas as well as discouraging high-risk behaviour such as taking selfies while driving may limit adverse events. “No selfie zones” are potentially dangerous spots in tourist areas that can be disastrous if public access is allowed. Many tourist areas in India, such as Nainital and Shimla, have sites such as sunset points, suicide points, and lovers’ points that are mountaintops or cliffs. These sites are associated with selfie-related injuries and deaths. Mumbai and Goa are pioneer cities in implementing “no selfie zones” [33]. This approach should be encouraged. Drawing the attention of social platform giants such as Facebook, Instagram, and Google to the risks of dangerous selfies, amending their current image upload policies so that such behaviour is not rewarded by the “likes” and “thumbs-ups” of fellow users and aggressively removing selfie challenge applications from app stores will potentially help save lives and prevent injuries.

The dangerous selfie, a vital link to selfie-related injuries and deaths, has been explored in this study, which is unique in the literature. The results of the study cannot be generalized to the young population, as the sample was limited to medical and nursing students. The possibility of recall bias cannot be excluded from this study. The self-report method, which may be prone to underreporting, was used for the assessment of the “dangerous selfie”. Some potential confounders, such as internet addiction and psychosocial factors, were not included in this study. We studied 595 participants, although the calculated sample size was 643. Detailed histories of selfie injuries were not collected. We also did not elicit a detailed history of how long participants had taken selfies daily or posted selfies on social media daily (the previous15 days or more was considered to avoid recall bias). For dangerous selfies, the criterion was whether the participants had ever taken one; however, for social media posting, it was whether they had posted for the previous 15 days or more.

## Conclusion

Nearly one in ten medical and nursing students reported taking dangerous selfies, and one in one hundred reported having been injured while attempting a selfie. Being male, posting selfies on social media daily and an SBS score > 75 were independent predictors of dangerous selfies. Further research is required to identify the community burden of dangerous selfies among the young population. Awareness should be created among students to prevent selfie-related injuries and fatalities. “No selfie zones” should be identified and implemented at high-risk sites in tourist areas.

## Supplementary information

**Additional file 1: Supplementary Table 1.** Selfitis Behaviour Scale (SBS) score distribution for participants . **Supplementary Table 2.** Association of “Dangerous Selfie” with SBS score (Quartile as assumptions).

## Data Availability

The dataset is available with corresponding authors and can be availed by request. This is not in the public domain due to author’s next project related to it.

## Abbreviations

AIIMS: All India Institute of Medical Sciences
AOR: Adjusted Odd Ratio
CI: Confidence Interval
MBBS: Bachelor of Medicine and Bachelor of Surgery
SBS: Selfitis Behaviour Scale
SD: Standard Deviation
