# Supplementary material for: Prevalence and determinants of the dangerous selfie among medical and nursing students: a cross-sectional study from eastern India
Source: BMC Public Health. 2020 May 6;20:628. doi: 10.1186/s12889-020-08785-4 (PMC7203966; doi:10.1186/s12889-020-08785-4)
Supplement: Supplementary file 1 — Additional file 1: Supplementary Table 1. Selfitis Behaviour Scale (SBS) score distribution for participants . Supplementary Table 2. Association of “Dangerous Selfie” with SBS score (Quartile as assumptions). [file 12889_2020_8785_MOESM1_ESM.doc]

**Supplementary Table 1: Selfitis Behaviour Scale (SBS) score distribution for participants**

| **Domain of Selfitis Behaviour Scale** | **Maximum score** | **Mean (SD)** |
| --- | --- | --- |
| Environmental Enhancement | 20 | 11.3 (3.8) |
| Social competition | 20 | 9.1 (3.5) |
| Attention seeking | 15 | 6.5 (2.6) |
| Mood modification | 15 | 6.8 (2.7) |
| Self- confidence | 15 | 6.9 (2.8) |
| Subjective conformity | 15 | 6.6 (2.7) |
| **Selfitis Behaviour Scale** | **100** | **47.1 (14.9)** |

**Supplementary Table 2: Association of “Dangerous Selfie” with SBS score (Quartile as assumptions)**

| **SBS Score**  **(0-100)** | **Total number of participants n=595** | **Total number of participants**  **“dangerous selfie”**  **n=52 (%)** | **Unadjusted odd ratio (95% CI)** | **P-Value** |
| --- | --- | --- | --- | --- |
| **0-25** | 56 | 5 (8.9) | Reference |  |
| **26-50** | 293 | 21 (7.2) | 0.79 (0.28-2.18) | 0.646 |
| **51-75** | 232 | 21 (9.1) | 1.02 (0.37-2.82) | 0.977 |
| **76-100** | 14 | 5 (35.7) | **5.67 (1.36-23.63)** | **0.017** |
